# Need of a revised and succinct atopic dermatitis diagnostic criteria for busy outpatient department in Nepalese context

**DOI:** 10.1002/ski2.468

**Published:** 2024-10-19

**Authors:** Prajwal Pudasaini, Sagar GC, Mauricio Salas‐Garza

**Affiliations:** ^1^ Dermatologist Department of Dermatology Civil Service Hospital Government of Nepal Kathmandu Nepal; ^2^ DEBRA Mexico Association Monterrey Mexico

## 1 INTRODUCTION

### 1.1 Background

Atopic dermatitis (AD) is the commonest chronic inflammatory skin disease. It is primarily a disease of childhood; however, its occurrence is not uncommon in adults.^1^ More than 80% of children are affected with this chronic condition by 5 years of age.^2^ A large proportion of the global population is affected by AD with a prevalence of 2.6% and its prevalence in Nepal varies from 6–7%.^3^

AD has an unpredictable course with frequent exacerbations in response to external stimuli and can be complicated by atopic march and neuropsychiatric implications.^4^, ^5^, ^6^, ^7^ Clinically, AD manifests with persistent, severely pruritic erythematous, oozing, papulae–plaque lesion over varied sites, depending on age, ethnicity and genetic background of those affected.

### 1.2 Prevalence and impact

For the purpose of clinical diagnosis, a variety of AD diagnostic criteria have been devised such as the classic Hanifin Rajka's criteria, the UK refinement of Hanifin and Rajka's criteria, Japanese Dermatological Association criteria, Korean Dermatological Association criteria, American Academy of Dermatology (AAD) consensus criteria and many more. All of the latter ones have been modified from the former—the classical Hanifin and Rajka's criteria— as per the need of the patients in their local context. Oftentimes, in countries such as Nepal with socio‐demographic, economic and cultural variations, these criteria become difficult to be implemented and there is a need of an inclusive, simple, yet a specific tool that caters to the clinical context of a local heterogenous group of Nepalese patients from across the country presenting to high‐volume centres.^8^

### 1.3 Existing diagnostic criteria

For decades, limitations to the classical Hanifin Rajka's criteria have been the long list of entities that need to be entailed for diagnosis and furthermore, it being time consuming, despite its high sensitivity. Similarly, the UK refinement criteria have its limitations, of being non‐inclusive as it does not incorporate the inclusion of patients more than 4 years of age, which has been a major backdrop, as up to 33% of adults can be affected with AD. To qualify as a case of AD with this criterion, it mandates that the child must have an itchy skin condition (or parental report of scratching or rubbing) plus three or more from the following: onset below age 2 years (not used if child is under 4 years), history of generally dry skin, history of skin crease involvement, personal or family history of other atopic disease such as the asthma or allergic rhinitis and/or visible flexural eczema.^9^

### 1.4 Challenges in the Nepalese context

Oftentimes, all the criteria cannot be fulfiled even in cases where clinicians have a strong clinical instinct for the diagnosis. For an instance, an adult AD patient with onset after 4 years of age with persistent pruritus, generally dry skin without flexural involvement, without skin crease involvement and personal history of atopy may not be fit as a clear case of AD; however, clinically it can be deemed as a case of AD by physicians' instinct.

A GA^2^LEN (Global Allergy and Asthma Excellence Network) Atopic Dermatitis Centre of Reference and Excellence (ADCARE) centre and a non‐profit government hospital that caters to more than 200 dermatology patients with heterogeneous skin diseases from across the country, our centre provides multitude of services in bare minimum cost to Nepalese patients. Eczemas including AD comprise one of the five most common skin diseases in our centre. However, more often than not, an easy diagnosis can be made with their typical phenotypic presentation, only occasionally. In the later cases, presentation can be misleading and misdiagnosis occurs especially with rampant use of multitude of treatment, including over the counter medications and home‐based remedies altering the phenotypic pattern of skin lesions.

Although a tertiary centre in the capital city, Kathmandu, services from our centre are restrained by diagnostic unavailability, high cost of treatment, lack of resources and lack of targeted therapies and biologics. Moreover, the availability of modern biologic medicines through compassionate supply programmes from abroad can only be foreseen if appropriate diagnosis is made, data are collected and therapeutic trials are performed in Nepalese context. In addition, government provision of AD targeted therapy can be sought for if appropriate data on prevalence and complication and its repercussion on patients and families are inferred. An appropriate diagnosis will lead to therapeutic trials to be performed in heterogenous population group from across the country and the result can be inferred for its usefulness in our context.

In line with this, to cater the unmet need and to grade the severity of AD in Nepalese patients, we have translated Patient Oriented Eczema Measure (POEM) in Nepalese language along with the translation and validation of the quality of life (QoL) tool—Skindex‐16 in Nepalese language.^10^

POEM was translated by following the standard protocol and after due permission from the copyright holder, the University of Nottingham, these translations were cross‐checked for accuracy by an expert dermatologist who had a good command over the English language and further back translation was checked.^11^

As one of the country's high‐volume centres that sees more than 200 dermatology patients, with a bare minimum time allocated to each patient due to time constraints, we have felt the need of a simple, concise yet a specific tool to diagnose and treat AD patients accurately in our resource‐poor busy OPD setting. Despite their seemingly apparent clinical complications and progression with atopic march and psychological concerns, oftentimes, diagnosis can be missed. Initial diagnosis can be skewed due to the lack of ability to fit into the complete set of criteria that have been designed for patients of different sociodemographic origin in the Europe and the Americas. Hence patients undergo multitude of insignificant treatments without diagnostic confirmation and/or treatment. This led to the idea of a revised diagnostic criteria for diagnosis in Nepalese context (Table 1). Resource‐poor high‐volume centres like ours pressed by constraints of time demands a more robust, brisk and succinct criteria inclusive for rapid and accurate diagnosis and hence a guide through therapy.

**TABLE 1:** Proposed new AD diagnostic criteria.

A) Localized [Must satisfy all 3 criteria's (1) (2) and (3) to diagnose as a case of localized AD, (±) criteria's (4) and (5)]
1) Persistent and/or recurrent *pruritus at any age*
2) Persistent and/or recurrent *asymmetric* involvement of ≤ *3 body parts* viz.
Lower legs
Ankles
Hands
Elbow flexures
Back
Calf
Behind the knees
3) Pruritus *not explained otherwise*:
No evidence of scabies (burrows/mites, eggs on dermoscopy)
No evidence of dermatophyte infections (KOH negative)
No evidence of allergic/Irritant contact dermatitis (patch test negative)
(±) with or without
4) Personal/Family history of atopiform diseases such as allergic rhinitis/asthma/atopic dermatitis)
5) Xerosis

B) Generalized [Must satisfy all 3 criteria's (1) (2) and (3) to diagnose as a case of generalized AD, (±) criteria's (4) and (5)]
1) Persistent and/or recurrent *pruritus at any age*
2) Persistent and/or recurrent *symmetric* involvement of > *4 body parts* viz.
Elbow extensors (bilateral)
Knee extensors (bilateral) in infants and children
Face
Back
Elbow flexures (bilateral)
Knee flexures (bilateral) in adults
Neck flexures
Eyes
3) Pruritus *not explained otherwise*:
No evidence of scabies (burrows/mites, eggs on dermoscopy)
No evidence of dermatophyte infections (KOH negative)
No evidence of allergic/Irritant contact dermatitis (patch test negative)
(±) with or without
4) Personal/Family history of atopiform diseases such as allergic rhinitis/asthma/atopic dermatitis)
5) Xerosis

Abbreviation: AD, atopic dermatitis.

### 1.5 Call to action

Henceforth, to address the unmet need and to compensate for the diagnostic lag, we call for action to encourage collaboration among healthcare professionals, researchers, policymakers and all the stakeholders to help develop and implement the proposed revised criteria best suited for resource‐poor high‐volume centres. We believe this could serve as evidence to our anecdote that fulfils our sole motive of overall improved patient outcomes in atopic dermatitis.

## CONFLICT OF INTEREST STATEMENT

The authors declare that they have no competing interests.

## AUTHOR CONTRIBUTIONS

**Prajwal Pudasaini**: Conceptualization (equal); data curation (equal); methodology (equal); project administration (equal); writing—original draft (equal); writing—review and editing (equal). **Sagar GC**: Formal analysis (equal); validation (equal); writing—original draft (equal). **Mauricio Salas‐Garza**: Conceptualization (equal); supervision (equal); validation (equal).

## ETHICS STATEMENT

Not Applicable.

## PATIENT CONSENT

Not Applicable.

## Data Availability

All data in this study are included in this published article.
